# Method and Analysis of High-Calcium Induced Differentiation of Human Primary Keratinocytes *In Vitro*

**DOI:** 10.3390/mps9050126

**Published:** 2026-08-28

**Authors:** Sarmistha Mahanty, Dharna Saini

**Affiliations:** Amity Institute of Biotechnology, Amity University Haryana, Gurugram 122413, India

**Keywords:** keratinocyte differentiation, high-calcium incubation, high-density differentiation, keratins, skin barrier, epidermis stratification

## Abstract

Epidermal development and regeneration rely on the stepwise differentiation of keratinocytes, the primary constituent cells of the epidermis. In the integrated epidermis, keratinocyte differentiation (KD) occurs in response to the increasing calcium gradient. Reflecting this, high-calcium incubation remains a popular approach for inducing KD *in vitro*. The outcome is primarily assessed by differential expression of differentiation markers compared to proliferative cells. However, it is significantly influenced by the cell models used, calcium concentration and incubation time, which often vary between studies. Such differences compromise reproducibility and comparability. In this article, we overview major parameters that shape the outcome of high-calcium induced KD. Comparative experimental data between high-calcium incubation and high-density induced differentiation underscore the need for combining additional parameters, such as microscopy based visualization with marker protein analysis for unbiased assessment of KD. These recommendations are intended to guide future research in keratinocyte biology.

## 1. Introduction

The epidermis is the outermost protective layer of the skin, containing most skin cells, performing most of the skin’s functions, and having the ability to regenerate. Thus, this layer is pivotal for maintaining skin health and hygiene. The epidermis is primarily constituted of keratinocytes, accounting for ~95% of epidermal cells, and is arranged into sublayers. It starts as a proliferative stem cell layer at the base of the dermis, called the ‘stratum basale’. Cells from this layer undergo a multi step differentiation program, creating distinct epidermal sublayers known as ‘stratum spinosum’, ‘stratum granulosum’, and terminally differentiated ‘stratum corneum’, representing the epidermal barrier (also known as ‘skin barrier’) [1,2,3,4]. Understanding the molecular processes underlying KD is thus critical for targeted modulation of skin health. The successful culturing of human epidermal keratinocytes on 3T3 feeder cells was first reported in 1975 by Rheinwald and Green, and later reinforced by Marcelo et al. [5,6]. Hennings and colleagues first demonstrated high-calcium (1.2 mM) induced differentiation of murine keratinocytes *in vitro*. Using calcium 1.2–1.8 mM, they further demonstrated the formation of desmosomes or cell-cell junctions within minutes of calcium addition and functional desmosomes within two hours [7,8]. Pillai et al. recapitulated these findings in human keratinocytes [9]. Menon et al. and others demonstrated the presence of an epidermal calcium gradient in the integrated epidermis and its role in epidermis stratification [10,11,12,13]. Later, the direct role of calcium induction was shown to be linked to differentiation marker expression [14] and in promoting lamellar body (LB) biogenesis. LBs are critical barrier organelles produced during the late stage of KD in the stratum granulosum layer [15,16,17]. Parallel mechanistic studies identified calcium receptors and downstream pathways, demonstrating that IP3-mediated PLCγ activation leads to high-calcium induced differentiation [18,19,20,21,22]. Additional signaling pathways, including JNK, MAP kinase, and Wnt, were implicated in the regulation of differentiation [23,24,25]. Parallel studies demonstrated vitamin D as a regulator of KD [26,27,28]. With its promising additive role, the use of vitamin D has surged in the cosmeceutical industry. Autophagy was later shown to be essential for KD, supporting survival in nutrient limited upper epidermis and enabling organelle removal during terminal stages, supporting cornification [29,30,31]. Overall, regulated by calcium signaling and autophagy, keratinocytes progress through differentiation stages with distinct marker expression, forming a cornified envelope and establishing a protective barrier [32,33,34,35]. These studies used mouse models, *in situ* human skin models, and *in vitro* keratinocyte cultures derived from human, murine, or canine sources, and differentiation was induced by varying calcium concentrations. Given that keratinocytes occupy defined sublayers according to their differentiation state, marker protein expression is consistent through epidermal sublayers *in vivo*. In contrast, in *in vitro* cultures, enrichment of a specific differentiation stage in the culture dish is influenced by multiple external factors, such as calcium concentration, incubation periods, and model systems [25,36,37]. These variables shape the conclusions of molecular studies [38]. Thus, a standardized protocol and analytical framework would guide future studies in the field.

## 2. Overview of Key Parameters

### 2.1. In Vitro Keratinocyte Cell Models

As stated above, the epidermis starts with a proliferative keratinocyte stem cell layer, called the stratum basale, which undergoes stepwise differentiation to generate the successive layers of the epidermis: the stratum spinosum, stratum granulosum, and stratum corneum. This process of establishing epidermal sublayers through layering of keratinocytes is termed epidermal stratification. Available keratinocyte cell models significantly differ in their stratification potential (summarized in Table 1) [36,37]. Neonatal human primary keratinocytes exhibit full stratification, meaning they can be differentiated into all three upper layers, as seen in the integrated epidermis. Importantly, primary keratinocytes closely preserve their native state, and thus the results are reproducible and comparable to *in vivo* conditions. For example, the distribution of acidic intracellular compartments (lysosomes) during primary keratinocyte differentiation was reported in 1992 and later in 2019 [39,40]. Similarly, reticular or fragmented Golgi apparatuses have been reported in integrated epidermis and in *in vitro* differentiated primary keratinocytes [40,41,42]. Finally, withdrawal of high calcium from differentiated culture causes excessive cell death [40], likely suggesting that differentiation commitment of primary keratinocytes is irreversible, as in integrated epidermis. Despite these advantages, primary keratinocytes are limited by short lifespan, high sensitivity to external factors, and difficulty in genetic manipulation. To address these challenges, immortalized models have been introduced. HaCaT cells, derived by spontaneous transformation, are widely used for molecular manipulation and CRISPR/Cas9 applications [43,44,45]. However, major limitations are that this cell line does not have full stratification potential and that HaCaT proliferation and differentiation states reversibly switch with the addition or removal of calcium, which is not true *in vivo* [45]. N/TERT-1 and N/TERT-2G cells, generated by hTERT upregulation and loss of P16INK4a, are reported to stratify into all three upper epidermal layers and are suitable for genetic manipulation, including CRISPR/Cas9-mediated deletion or expression of proteins [46,47,48]. Thus, N/TERT cells are expected to significantly overcome the limitations of both primary keratinocytes and HaCaT cells. More recently, iPSCs have emerged as an effective alternative due to their unlimited proliferation capacity and modeling of epidermal diseases, keeping the genetic background the same [49,50,51]. However, major challenges include the extended time required for culturing and the associated cost. Overall, careful selection of cell type aligning with the experimental purpose is essential.

### 2.2. Markers of Keratinocyte Differentiation

**Figure 1:**
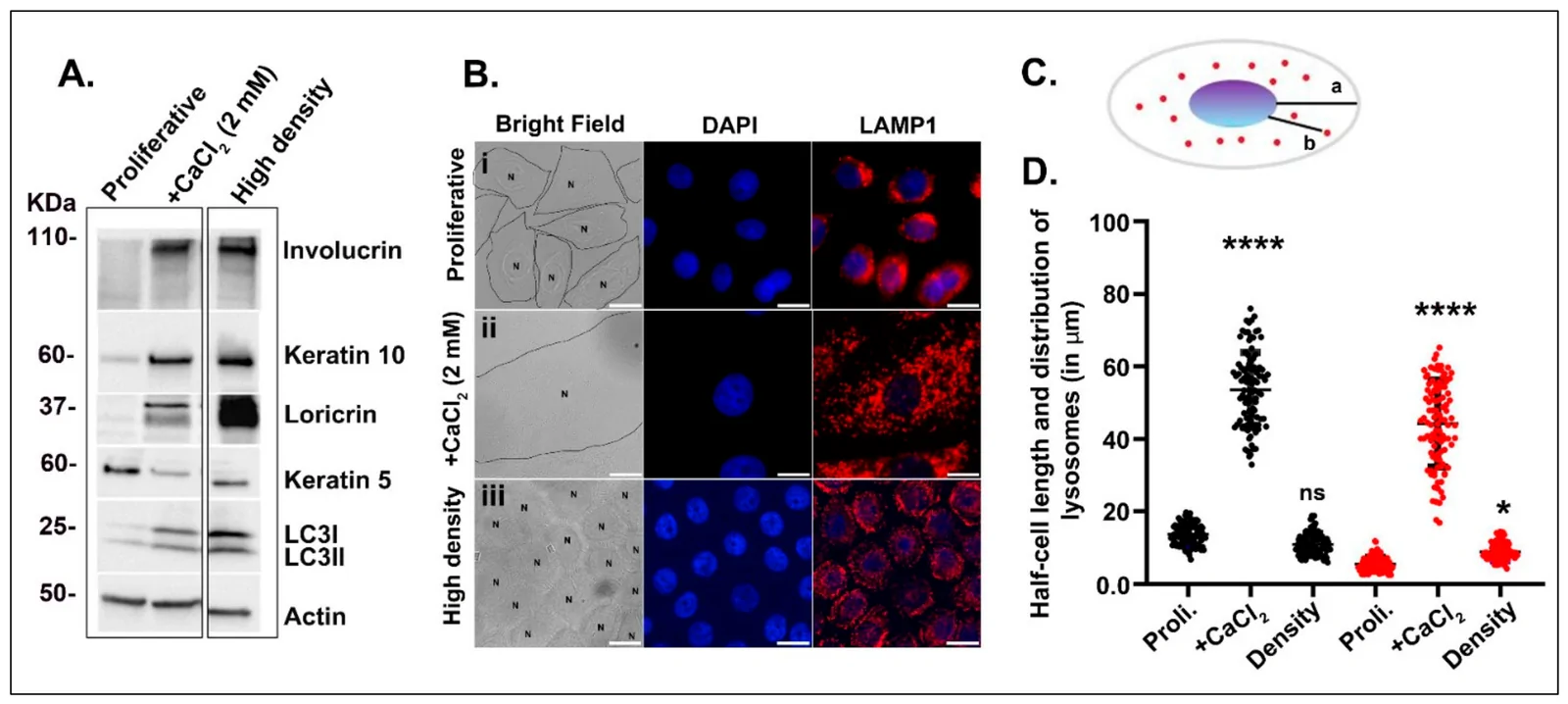
(**A**). Both high-calcium induction and high-density lead to differential expression of marker proteins compared to proliferative keratinocytes. Involucrin, Keratin 10, Loricrin, and LC3I/II were taken as differentiation markers, while Keratin 5 was included as a proliferative marker. These experiments were performed in three independent biological replicates. (**B**(**i**)). Morphology of proliferative keratinocytes (passage 3) and perinuclear-restricted lysosomal structures. (**B**(**ii**)). Keratinocytes differentiated by high-calcium incubation (2 mM CaCl_2_) exhibit a significant increase in cell area and redistribution of lysosomes throughout the cytoplasm compared to proliferative keratinocytes. (**B**(**iii**)). High-density induced keratinocytes retain a cell size and lysosomal distribution pattern similar to proliferative cells. Scale bar = 10 μm. N = nucleus. Please note that these experiments were performed in multiple biological and technical replicates (≥3). (**C**). Schematic representation of the measurement of half-cell length (‘a’) and lysosomal distribution as a distance from the nucleus (‘b’). (**D**). The graph displays half-cell length measured from bright-field images (as black circles) and lysosomal distribution using immunofluorescence images (as red circles) measured by drawing a straight line after setting the scale in microns in Fiji software. The quantification involves 100 cells of each category representing multiple independent experiments (biological replicates). Data are presented as mean ± SD, with SD reflecting variability within each group. Statistical analysis was performed using an F-test to compare variance between proliferative versus +CaCl_2_ and proliferative versus high-density differentiation groups. **** *p* ≤ 0.0001; * *p* ≤ 0.05; ns = non-significant.

Keratinocytes produce approximately 58 distinct keratins, along with other structural proteins and enzymes that form the cornified envelope at terminal differentiation [52,53]. Due to their stage specific expression, these structural proteins serve as reliable markers of differentiation. For instance, proliferative stem cells express keratin 14 and keratin 5; early differentiating cells express involucrin, keratin 1, and keratin 10; and late stage cells express loricrin and filaggrin [4,54]. Specialized structures such as desmosomes arise during differentiation, and proteins such as cadherins/plaque proteins that label these structures are additional markers of KD. In *in vitro* cultures, as differentiated cells increase, the percentage of proliferating cells decreases proportionally. Therefore, with increased expression of differentiation markers such as keratin 1/10 (as in Figure 1A), a decrease in proliferative markers such as keratin 5/14 is expected and should be included as an analytical parameter. Moreover, proliferative multipotent stem cells are columnar and 6–12 µm in diameter, and lysosomes are the most abundant organelles localized perinuclearly. Under high-calcium incubation, cell size expands to 60–65 µm, and lysosomes redistribute throughout the cytoplasm (Figure 1B(ii),D) [40,55,56]. These phenotypic changes are easily detectable using immunofluorescence microscopy and will help overcome the limitations of biochemical interpretation [40,41,57,58]. As shown in Figure 1B(iii), while high-density induces differentiation marker protein expression, the cellular characteristics are significantly different from those of calcium-incubated keratinocytes or those defined *in vivo*.

### 2.3. Method of Keratinocyte Differentiation

Following the *in vivo* role of the epidermal calcium gradient, high-calcium incubation remains the most widely used method for KD *in vitro* [4,59,60,61]. Extracellular calcium is sensed through calcium sensing receptors (CaSR), increasing the levels of IP3 and DAG, which respectively release Ca^2+^ from the ER calcium store and activate the phospholipase C (PLC) pathway, triggering downstream signaling pathways promoting differentiation [18,62]. Proliferative keratinocyte culture medium contains a physiological calcium concentration of approximately 50–60 μM. To induce differentiation, calcium chloride (CaCl_2_) is added to the culture medium. Reported concentrations of CaCl_2_ used for differentiation range between 0.5 mM and 2 mM, with incubation times varying from 24 h to 12 days. These variations may contribute to the percentage of differentiated cells and thus the differentiation marker expression [63,64,65,66,67,68,69]. Therefore, experimental outcomes may not accurately reflect tested conditions, such as genetic or chemical manipulation, but rather be influenced by differentiation conditions themselves. Passage number and cell density may also significantly influence KD. Since the proliferative capacity of primary cells declines with successive passages, and since KD is closely linked to cell cycle exit, a comparatively higher passage (p3-p4) would favor more differentiated cells in the population than those at lower passage (p1-p2) [63,70,71]. Moreover, as differentiated cells increase in size, higher confluence would limit cellular expansion and thus would impact differentiation. When reported, a confluence of 60–65% is considered optimal for inducing the differentiation process [40,72]. To achieve a more homogeneous population in an *in vitro* culture, serum supplementation, alongside calcium, has been proposed to additively activate calcium-PLC signaling [38]. Loss of adherence, as in suspension culture, can also induce KD, but the major limitation is that rounded cells in suspension prevent analysis of morphological and organelle features and are also not comparable to the *in vivo* condition, where cells are typically not rounded [73]. High-density induced differentiation is another well known approach that induces expression of late differentiation markers such as loricrin or LB related proteins. However, cellular characteristics closely resemble those of proliferative keratinocytes and not calcium induced differentiated cells (Figure 1B(i–iii). Nonetheless, the expression of late differentiation markers in high-density cultures is likely induced by cell-cell junctions [35,72,74,75,76]. Collectively, while calcium induced differentiation remains the most suitable approach, the associated parameters described above should be considered.

## 3. Experimental Design

### 3.1. Materials

Primary neonatal human keratinocytes (Lonza, Basel, Switzerland, Cat#00192907) were cultured using the KGM^®^ Keratinocyte Growth Medium BulletKit^®^ Culture System (Lonza, Cat#CC-3111). 1X PBS (Gibco, Thermo Fisher Scientific, Waltham, MA, USA, Cat#14040117), rat collagen Type I (Sigma-Aldrich, St. Louis, MO, USA, Cat#C3867), trypsin-EDTA (Gibco, Cat#15090046), trypsin-neutralizer (Gibco, Cat#R002100), and calcium chloride (Sigma-Aldrich, Cat#C5670) were used as required. Primary antibodies included LAMP1 (Developmental Studies Hybridoma Bank, Iowa City, IA, USA, Cat#H4A3), LC3A/B (Cell Signaling Technology, Danvers, MA, USA, Cat#4108), β-actin (Cell Signaling Technology, Danvers, MA, USA Cat#4967), keratin 5 (Invitrogen, Thermo Fisher Scientific, Waltham, MA, USA Cat#PA532465), keratin 10 (Invitrogen, Cat#PA597907), loricrin (Invitrogen, Cat#PA530583), and involucrin (Santa Cruz Biotechnology, Dallas, TX, USA, Cat#(A-5): sc-398952). Tissue culture plastics were obtained from Corning (Corning, NY, USA) and BD Biosciences (San Jose, CA, USA).

### 3.2. Equipment

The equipment included: BSL-2 cell culture system (Thermo Fisher Scientific, Waltham, MA, USA; 1300 Series A2, Model 1384); CO_2_ humidified incubator (Thermo Fisher Scientific, Waltham, MA, USA; Forma Steri-Cycle, Model 3.71); Eppendorf centrifuge (Eppendorf AG, Hamburg, Germany; Model 5810-R); and small laboratory equipment such as micropipettes, glass slides, coverslips, and forceps. Microscopy was performed using a Dragonfly 400 confocal microscope equipped with an Andor SONA sCMOS camera (Andor, Oxford Instruments, Belfast, UK) and an Olympus IX81 motorized inverted fluorescence microscope equipped with a CoolSNAP HQ2 (Photometrics) CCD camera (Olympus Corporation, Tokyo, Japan).

## 4. Procedure

### 4.1. High Calcium-Induced Differentiation

**A.**  **Cell Source, Storage and Culture Medium:**

Neonatal human primary keratinocytes were obtained commercially from Lonza (Cat#00192907) and cryopreserved in liquid nitrogen until thawed.

Note: Cells were cultured in the recommended medium, the KGM^®^ Keratinocyte Growth Medium BulletKit^®^ Culture System (Cat#CC-3111), for proliferation. Each kit contains: Basal Medium-500 mL (calcium-free formulation) that needs to be supplemented with the components provided in separate vials in the kit; Hydrocortisone-0.50 mL; Transferrin-0.50 mL; Epinephrin-0.25 mL; GA-1000 (Gentamicin/Amphotericin B mix)-0.50 mL; Bovine Pituitary Extract (BPE)-2.00 mL; Human Epidermal Growth Factor (hEGF)-0.50 mL; and Insulin-0.50 mL. Culture medium was reconstituted as per the manufacturer’s instructions.

To induce differentiation, CaCl_2_ (Sigma-Aldrich, Cat#C5670) was added to this reconstituted culture medium at a final concentration of 2 mM (from a stock solution of 0.1 M in H_2_O).

**B.**  **Collagen Coating of Culture Flasks (Optional Step):**

A cryovial containing Neonatal Normal Human Epidermal Keratinocytes (NHEK-Neo) containing ≥500,000 cells were thawed in a T75 flask (75 cm^2^ growth area). The flask was coated with collagen (Sigma-Aldrich, Cat#C3867) to enhance cell adherence.

B-i. The working collagen solution (50 µg/mL) was prepared in 1X PBS containing 0.02 N acetic acid. Approximately 4 mL of the collagen working solution was added to a T75 tissue culture flask in the hood. The flask was incubated for 30 min at room temperature (RT) with intermittent swirling to ensure uniform coating.

B-ii. Removal of Collagen and Preconditioning of Flask

After 30 min, the collagen solution was aspirated, and the flask was rinsed twice with sterile 1X PBS. Subsequently, 15 mL of culture medium was added to the flask, which was then placed in a humidified incubator at 37 °C with 5% CO_2_.

Note: This collagen-coating step is optional. For BD Falcon™ T75 flasks (BD Biosciences, Cat#353136), coating is not mandatory. In such cases, the flask surface can be briefly rinsed with 1X PBS, followed by the addition of 15 mL of culture medium prior to incubation.

**C.**  **Thawing of Cryopreserved Cells:**

C-i. A cryovial containing keratinocytes was retrieved from liquid nitrogen storage and thawed in a preheated 37 °C water bath for approximately 40 s until only a small ice crystal remained.

C-ii. The thawed cells were mixed with 4 mL of culture medium that was preheated to 37 °C.

C-iii. The whole content (5 mL) was then transferred to the prepared T75 flask containing 15 mL culture medium and incubated in the humidified incubator at 37 °C with 5% CO_2_.

C-iv. The culture medium was changed within 24 h. Then, it was changed every 48 h until it reached a confluence of about 75–80%.

**D.**  **Detachment of cells and subculturing:**

D-i. The culture medium was aspirated, and the dish was briefly washed with 4 mL of 1X PBS. Cells were detached using 2 mL of trypsin-EDTA (Gibco, Cat#15090046) and incubated for 5–7 min with gentle swirling in a humidified incubator at 37 °C with 5% CO_2_.

D-ii. Once detached, an equal volume (2 mL) of trypsin neutralization solution (Gibco, Cat#R002100) was added, and the suspension was mixed by gentle pipetting (using a 10 mL serological pipette) and transferred to a 15 mL Falcon tube.

D-iii. Cells were centrifuged for 3 min at 2500 rpm at RT, resuspended in 1 mL culture medium, and gently mixed using a 1 mL pipette.

D-iv. A total of 20 µL of this suspension was mixed with trypan blue (Gibco, Cat# 15250061) (1:1 ratio), and cells were counted using a hemocytometer under a brightfield microscope with a 10× or 20× objective.

**E.**  **Seeding for Experimental Assays:**

To achieve ~60% confluence the following day, (a) 4 × 10^5^ cells were seeded in a well (35 mm) of a 6 well plate containing collagen coated glass coverslips for immunofluorescence, or (b) 1 × 10^6^ cells on a 60 mm collagen coated dish for immunoblotting.

**F.**  **Induction of Differentiation:**

F-i. At ~60% confluence, the culture medium was replaced with high-calcium medium supplemented with 2 mM CaCl_2_ from a stock solution of 0.1 M (please refer to Section 4.1: A ‘Cell Source, Storage and Culture Medium’), and cultures were incubated in a humidified incubator at 37 °C with 5% CO_2_.

F-ii. High-calcium medium was replenished every 24 h for a total of 3 days (72 h).

F-iii. **Optional** Condition

To obtain a more homogeneous population, 2% FBS may be added along with 2 mM CaCl_2_. This step was not included in the experiments described herein.

**G.**  **Downstream Analyses:**

G-i. For immunofluorescence:

At the end of incubation, cells on coverslips were fixed with 4% paraformaldehyde (Sigma-Aldrich, Cat #158127) for 20 min at RT and washed three times with 1X PBS (2 min each). Coverslips were then simultaneously permeabilized and immunostained in antibody solution containing 0.2% saponin (Sigma-Aldrich, Cat#84510), 0.1% BSA (Sigma-Aldrich, Cat #A9647), and anti-LAMP1 antibody in 1:200 dilution at RT for 40 min. Coverslips were washed with 1X PBS and incubated with Alexa Fluor A594 conjugated secondary antibody for 20 min. Finally, coverslips were washed in 1X PBS and mounted on glass slides (see Methodology for further details). Cells were visualized under an Olympus IX81 motorized inverted fluorescence microscope equipped with a CoolSNAP HQ2 (Photometrics) CCD camera and a Dragonfly 400 confocal microscope (Andor, Oxford Instruments) equipped with an Andor SONA sCMOS camera.

G-ii. For Immunoblotting:

Cells cultured in 60 mm dishes were scraped using a cell scraper (Corning, Cat #CLS3011) in 1X PBS and pelleted by centrifugation (3000 rpm for 5 min). Scraping is preferred over trypsinization because it maintains differentiated cells in their original state. Trypsinization, on the other hand, may separate the cells by distorting junctional structures. Cell lysates were prepared in lysis buffer containing 1% Triton X-100, 1 mM EDTA, 150 mM NaCl, and 20 mM Tris (pH 7.4), and were subjected to immunoblotting analysis as described previously [40], with specific details as below.

### 4.2. High-Density Induced Differentiation

H. For density induced differentiation, Steps A-E were performed exactly as described for the high-calcium condition.
I. After reaching ~95% confluence, cells were cultured for an additional 3 days, with medium replenished every 24 h.
J. The rest of the procedures for downstream analysis were followed exactly as in step-G above.

## 5. Experimental Methods and Data Analysis

Immunostaining and immunoblotting:

Immunostaining was performed as previously described [41]. Briefly, 4% PFA-fixed cells were simultaneously permeabilized and immunostained in antibody solution containing saponin (0.2%) (Sigma-Aldrich, Cat#84510), 0.1% BSA (Sigma-Aldrich, Cat#A9647), and anti-LAMP1 antibody (DSHB, Cat#H4A3) in 1:200 dilution at RT for 40 min. Coverslips were gently washed with 1X PBS and incubated with Alexa Fluor A594 conjugated secondary antibody (Thermo Fisher Scientific, Cat#A11032) in 1:500 dilution for 30 min at RT. Coverslips were gently washed with 1X PBS and mounted using antifade mounting medium with DAPI (Thermo Fisher scientific, Cat#P36962). This experiment was performed in at least three biological replicates and multiple technical replicates.

For immunoblotting, cell pellets were lysed using lysis buffer containing 1% Triton X-100, 1 mM EDTA, 150 mM NaCl, and 20 mM Tris (pH 7.4) with a protease inhibitor cocktail (Roche, Basel, Switzerland, Cat#4693116001). The tubes were incubated on ice for 30–40 min with intermittent tapping. Tubes were spun at 12,000 rpm for 5 min, and the supernatant was collected. Protein estimation was done using Pierce™ BCA Protein Assay Kit (Thermo Fisher Scientific, Cat#A65453) and quantified against a standard curve as per the manufacturer’s guidelines. A total of 15 µg of protein was loaded onto precast Western blot gels, followed by transfer to nitrocellulose membrane (BIORAD, Hercules, CA, USA, Cat#4561084 and, BIORAD, Cat #1704158). The blots were incubated overnight with primary antibodies in 1:1000 dilutions made in 5% non-fat milk in 1X PBST (1X PBS with 0.1% Tween 20) at 4 °C. Post incubation, blots were washed with PBST and incubated with HRP-conjugated secondary antibodies (Abcam, Cambridge, UK, Cat#ab6721 and Cat#ab6789) in 1:5000 dilutions and incubated for 2 h at RT on a platform rocker. The blots were washed with 1X PBST at least twice (5 min each) and developed using the Clarity Western ECL Substrate (BIORAD, Cat#1705060) and Western blotting detection system (BIORAD, ChemiDoc MP). These experiments were done in three biological replicates.

Data Analysis in ImageJ/Fiji (version: 1.54p): The half-cell length (µm) was measured by drawing a straight line from one edge of the nucleus to the respective apical end of the cell. Similarly, lysosomal distribution (µm) was measured by drawing a line from one edge of the nucleus to the most distant LAMP1-positive compartment on the same apical side of the cell (schematically represented in Figure 1C). Independent values were plotted as mean ± SD, and statistical significance was calculated using GraphPad Prism, version 6.04.

## 6. Result and Conclusions

As shown in Figure 1, high-calcium incubated cells are significantly larger in size, display dispersed LAMP1-positive lysosomes, and show increased expression of differentiation markers involucrin, keratin 10, and LC3I/II, along with a concomitant decrease in the proliferation marker keratin 5 (Figure 1A,B,D), confirming differentiation. Likewise, while in high-density differentiation expresses differentiation markers such as loricrin, cellular characteristics are significantly different from those of high-calcium incubated cells. In line with this, it has been previously described that expression of late differentiation markers can be triggered through enhanced cell-cell contact in high-density conditions. Two important conclusions can be made from this particular observation: (1) The calcium induced differentiation method is the most reliable and suitable method as it mimics most features of *in vivo* epidermis; and (2) the interpretation of *in vitro* differentiation solely based on marker protein expression could be misleading, and additional visual parameters such as cell size and organelle distribution need to be included. Overall, careful consideration of cell type, differentiation method, and analytical parameters is essential for reliable assessment of experimental studies on KD.

## Figures and Tables

**Table 1:** Comparison of available keratinocyte cell models in terms of their stratification potential, advantages, and limitations. The studies cited in the Table correspond to the original publications that first introduced each respective cell model. For detailed descriptions, please refer to the main text.

Cell Model	Stratification	Advantages	Limitations	References (First Introduced)
**Primary Keratinocytes**	Can stratify into all epidermal layers *in vitro* and in organotypic cultures.	Excellent fidelity. Physiologically relevant.	Short lifespan. Technically demanding culture conditions. Difficult for genetic manipulation.	[5,7,8]
**HaCaT**	Reduced stratification *in vitro*. Stratifies in organoid models.	Easy to culture. Easy to manipulate. Widely available and standardized.	Aneuploid genome. Altered signaling. Reduced fidelity compared to primary cells.	[43,44]
**N/TERT-1/N/TERT-2G**	Stratifies into all epidermal layers with functional barrier in organotypic models; closer to primary keratinocytes.	More physiologically relevant than HaCaT. Easier to manipulate.	Requires genetic modification (hTERT + p16 bypass) that may alter subtle pathways. Less widely adopted. Culture variability.	[46,47,48]
**iPSCs**	Can differentiate into keratinocytes. Theoretically, full stratification potential.	Unlimited expansion. Patient-specific. Powerful for rare disease modeling and regenerative medicine.	Low efficiency. Risk of genomic instability and tumorigenicity. Technically complex. Costly.	[49,50,51]

## Data Availability

The original contributions presented in this study are included in the article. Further inquiries can be directed to the corresponding author.
